# Petroleum degradation by *Pseudomonas* sp. ZS1 is impeded in the presence of antagonist *Alcaligenes* sp. CT10

**DOI:** 10.1186/s13568-018-0620-5

**Published:** 2018-05-28

**Authors:** Jibei Liang, Tao Cheng, Yi Huang, Jianhua Liu

**Affiliations:** 1 Ocean College, Zhejiang University, Marine Science Building #379, Zhoushan Campus, 1 Zheda Road, Dinghai District, Zhoushan, 316000 ZJ China; 2 Ocean Research Center of Zhoushan, Zhejiang University, Zhoushan, 316021 ZJ China

**Keywords:** *Alcaligenes* sp., Antagonism, Bioremediation, Cyclodipeptide, Oil-degrading microorganism, *Pseudomonas* sp.

## Abstract

Enhanced bioremediation is a favorable approach for petroleum pollutant cleanup, which depends on the growth of oil-eating microorganisms. In this study, we show that, by using the modified T-RFLP (mT-RFLP) methodology, one of the four major microbial populations derived from oil sludge has failed to propagate in MS medium supplemented with 2% yeast extract (YE). rDNA sequence-based analysis indicated that the four populations were *Donghicola* sp. CT5, *Bacillus* sp. CT6, *Alcaligenes* sp. CT10, and *Pseudomonas* sp. ZS1. Four purified strains grow well individually in MS medium supplemented with 2% YE, suggesting that ZS1 growth is antagonized by other strains. Co-growth analysis using mT-RFLP methodology and plate inhibitory assay indicated that ZS1 exhibited antagonistic effect against CT5 and CT6. On the other hand, co-growth analysis and plate inhibition assay showed that CT10 antagonized against ZS1. To investigate the potential compounds responsible for the antagonism, supernatant of CT10 culture was subjected to GC–MS analysis. Analysis indicated that CT10 produced a number of antimicrobial compounds including cyclodipeptide c-(L-Pro-L-Phe), which was known to inhibit the growth of *Pseudomonas* sp. Growth test using the purified c-(L-Pro-L-Phe) from CT10 confirmed its inhibitory activity. We further showed that, using both gravimetric and GC analysis, CT10 antagonism against the oil-eating ZS1 led to the diminishing of crude oil degradation. Together, our results indicate that bioremediation can be affected by environmental antagonists.

**Electronic supplementary material:**

The online version of this article (10.1186/s13568-018-0620-5) contains supplementary material, which is available to authorized users.

## Introduction

Petroleum leakage is a major threat to land and marine environment (Holliger et al. 1997). Physical methods involving removal of solid and liquid pollutants are tedious and expensive; and chemical methods using chemically synthesized surfactants can cause secondary pollution (Kanaly and Harayama 2010; Murphy et al. 2005). Enhanced bioremediation method using indigenous oil-eating microorganisms and biosurfactants is believed to be a favorable method for oil spill cleanup (Patowary et al. 2016; El-Bestawy et al. 2014; Karamalidis et al. 2010; Al-Mailem et al. 2017; Varjani and Upasani 2016).

Many studies have focused on the physical and chemical conditions at the pollution sites that affect the performance of enhanced bioremediation (Díaz-Ramírez et al. 2003; Venosa and Zhu 2003). Physical conditions such as temperature and salinity affect the growth of many microorganisms. Similarly, chemical conditions include mineral salts and pollutant toxic compounds also influence the growth of various microorganisms. Hence, indigenous isolates of microorganisms are advantageous for enhanced bioremediation (Patowary et al. 2016; El-Bestawy et al. 2014). However, it remains unclear whether antagonism between microorganisms including the oil-eating ones will affect the cleanup of oil pollutants.

Antagonisms between microorganisms are not uncommon. Many antibiotics are discovered through the observation of compounds that are nontoxic to humans but exhibit antagonistic effect against pathogenic microbes. The most well-known example is the discovery of penicillin by Fleming (Fleming 1929). Before long, Waksman has established the plate inhibitory method for systematic screening for soil microbes, especially the *Actinomyces* spp. that are capable of inhibiting pathogenic microbes (Waksman and Woodruff 1940). A number of antibiotics were successfully identified and characterized including streptomycin and neomycin that have extensively been applied to the treatment of numerous infectious diseases (Waksman and Woodruff 1940).

Restriction fragment length polymorphism (or RFLP) analysis of amplified rDNA allows identification of microorganisms such as mycobacterium species (Vaneechoutte et al. 1993). Terminal fluorescence labeled RFLP (T-RFLP) analysis is a method for identification of mixed microbial populations with the help of DNA sequencer (Liu et al. 1997). We have previously modified the T-RFLP (mT-RFLP) method by replacing DNA sequence gel with mini-PAGE gel to study dynamic change of microbial populations without the need for DNA sequencer, an equipment uncommon in many biology laboratories (Cheng et al. 2017). By using the mT-RFLP analysis, we have isolated the rhamnolipid-producing oil-eating *Pseudomonas* sp. ZS1 strains from the mixed culture of petroleum sludge-originating microbes cultivated in MS medium supplemented with 2% glucose (Cheng et al. 2017).

In this study, we show that the growth of *Pseudomonas* sp. ZS1 is suppressed in the mixed culture of sludge-originating microbes in medium without glucose. Co-growth and plate inhibition analyses reveal that an *Alcaligenes* sp. CT10 strain exhibits antagonistic effect against *Pseudomonas* sp. ZS1. GC–MS analysis shows that a number of antimicrobial compounds including cyclodipeptide c-(L-Pro-L-Phe) present in supernatant of CT10 culture. Both gravimetric and GC analyses show that CT10 impedes the oil-degradation by ZS1, implying that antagonisms between environmental microorganisms can affect the outcome of bioremediation.

## Materials and methods

### Strains, DNA, and cultures

Petroleum sludge was collected in April 2016 at Sanjiang Ferry Terminal, Zhoushan, Zhejiang province, China. Microbial strains were resuspended and maintained in mineral salt (MS) medium (1 L contains: 0.6 g Na_2_HPO_4_, 0.2 g KH_2_PO_4_, 4.0 g NaNO_3_, 0.3 g MgSO_4_, 0.01 g CaCl_2_, 0.01 g FeSO_4_, 1 g or 0.1% yeast extract or YE) (Zajic and Supplison 1972). For propagation, strains were cultivated in glass conical flask at 30 °C in MS medium supplemented with 2% YE or 1% crude oil. Cell growth was monitored by either colorimetric (optical density at the wavelength of 600 nm) or gravimetric methodologies (cell dry weight). All measurements were performed in triplicate, unless otherwise stated.

*Pseudomonas* sp. ZS1, *Alcaligenes* sp. CT10, *Donghicola* sp. CT5 and *Bacillus* sp. CT6 strains were deposited in the China General Microbiological Culture Collection Center with the accession numbers of CGMCC-13460, CGMCC-1.16509, CGMCC-1.16485 and CGMCC-1.16486 (respectively) and whose 16S rDNA sequences were deposited in NCBI GenBank with the accession number of KY437088, KY437091, KY437089 and KY437090, respectively.

Oligonucleotide DNA sequences 27F, 5′-AGAGTTTGATCMTGGCTCAG-3′ and 1492R, 5′-TACGGYTACCTTGTTACGACTT-3′ (Moreno et al. 2002) used in PCR amplification of 16S rDNA were purchased from BGI (BGI, Shenzhen, China).

### Preparation of genomic DNA and PCR analysis

To obtain microbial genomic DNA for PCR amplification, mixed or clonal microbial cultures were pelleted by centrifugation and the resulting pellet was resuspended in lysis solution using Genomic DNA Extraction kit (Axygen Scientific Inc., Tewksbury, MA, USA) and extracted according to the manufacturer’s instruction. The 16S rDNA fragment was PCR amplified by using the microbial genomic DNA as template and 16S rDNA-specific primers 27F and 1492R (Moreno et al. 2002). The PCR condition was set as follows: after the initial denaturation at 95 °C for 5 min, 30 cycles of 95 °C for 30 s, 55 °C for 30 s, and 72 °C for 90 s, and a final extension at 72 °C for 10 min. The resulting PCR fragment was subjected to sequencing analysis in BGI (BGI, Shenzhen, China) and compared with NCBI’s nucleotide sequences using BLAST tools (http://www.ncbi.nlm.nih.gov).

### Modified T-RFLP or mT-RFLP analysis

To examine the dynamic change of mixed microbial populations under various growth conditions, we modified the T-RFLP method (Liu et al. 1997) by using the mini-PAGE gel instead of sequencing gel. In brief, the 16S rDNA fragments were PCR amplified on genomic DNA as template derived from microbial populations using the 27F-fluorescence labeled and 1492R unlabeled primers. The resulting fragments were subjected to *Hha*I (New England Biolabs Inc., Ipswich, MA, USA) digestion and 8% mini-PAGE gel electrophoresis. Fluorescence signals were captured using the Gel Imaging System Tanon 5200 (Tanon Scientific Inc., Shanghai, China) with a SybrGreen fluorescence channel.

### Co-growth assay

Equal amount of overnight cultures was mixed and inoculated to fresh medium to a concentration of 0.1 OD_600_. Cell populations at various time points during growth were examined using mT-RFLP analysis (see above). Gel image was recorded using the Gel Imaging System Tanon 5200 (Tanon Scientific Inc.,).

### Preparation of supernatant crude extract for growth inhibition analysis

Growth inhibitory factors in cell-free supernatant of *Pseudomonas* sp. ZS1 and *Alcaligenes* sp. CT10 cultures were prepared by following the methods previously reported by Zhang et at. (Zhang and Miller 1992) and Bharali et al. (Bharali et al. 2011), respectively. In brief, supernatant of ZS1 culture was acidified to pH 2.0 using HCl. The resulting precipitate was collected by centrifugation at 13,400*g* for 30 min and dissolved in bicarbonate (pH 8.6) and extracted twice with chloroform-ethanol (2:1 v/v) solution. The organic phase was evaporated and the resulting paste or crude extract was used in growth inhibition assay. Likewise, supernatant of CT10 culture was acidified to pH 2.0 using HCl and kept at 4 °C overnight. The turbid supernatant was extracted twice with an equal volume of ethyl acetate and collected through a separating funnel. Subsequently, the organic phase was evaporated and the resulting paste or crude extract was used in growth inhibition test and GC–MS analysis for bioactive compounds.

### Plate inhibitory assay

To examine the growth inhibitory activities of the supernatant, crude extract (see above) of supernatants was dissolved in chloroform to a final concentration of 50 mg mL^−1^. Filter discs containing 20 μL 50 mg mL^−1^ supernatant crude extract were placed on top of MS agar plates that were inoculated with the test strains. As control, filter discs containing 20 μL solvent chloroform and 20 μl 50 mg mL^−1^ ampicillin in water were also placed on the same plate. Images were taken 1–3 days after incubation at 30 °C.

### GC–MS analysis of compounds extracted from supernatant of *Alcaligenes* sp. CT10 culture

The compounds extracted from supernatant of *Alcaligenes* sp. CT10 culture (CEAC) was analyzed by gas chromatography coupled with mass spectrophotometer (GC–MS). 1 µL of CEAC was directly injected into the injection port of gas chromatograph (Shimadzu 2010Plus GC system, Shimadzu Co., Tokyo, Japan) coupled with a mass spectrometer system (MS) (Shimadzu QP2020 with quadrupole analyzer). The GC was operated on an Rtx-5MS GC column (30 m × 0.25 mm, id. with 0.25 µm film thickness of 5%-phenyl-methylpolysiloxane) (Restek Co., Bellefonte, PA, USA) and helium (purity 99.999%) was used as the carrier gas. The temperature of the injection port was set to 250 °C while the sample injection was made in splitless mode with a purge flow 50 mL min^−1^ for 1 min. The temperature program was started with an initial temperature at 50 °C and held for 2 min at this temperature, then 6 °C min^−1^ to 300 °C for 20 min a flow rate of 1 mL min^−1^ and run time 63.67 min. The mass spectrometer was operated in electron ionization (EI) mode with the ion source temperature at 230 °C. The MS quad temperature was set at 150 °C. The electron energy was 70 eV. Full-scan MS data were acquired in the range of 50–500 m/z to obtain the fragmentation spectra of CEAC. The LabSolutions (Shimadzu Co.) was used to determine all the peaks in raw GC chromatogram. Library search was done for all the peaks using the National Institute of Standards and Technology NIST/EPA/NIH (NIST 14 Library). All results were combined into a single peak table (Table 1).

**Table 1:** Compounds derived from supernatant of *Alcaligenes sp.* CT10 cultures

No.	RT^a^	Compound name	M.W.	Formula	%Pk^b^	Comment^c^
1	5.44	Ethylbenzene	106	C_8_H_10_	0.86	–
2	5.63	1,4-Dimethylbenzene	106	C_8_H_10_	2.35	–
3	5.67	1,3-Dimethylbenzene	106	C_8_H_10_	0.7	–
4	6.12	Styrene	104	C_8_H_8_	5.86	–
5	11.87	*n*-Hendecane	156	C_11_H_24_	6.76	–
6	15.66	2(3H)-benzofuranone	134	C_8_H_6_O_2_	2.79	Insecticidal Fan et al. (2008)
7	16.15	Benzeneacetic acid	136	C_8_H_8_O_2_	34.54	Antimicrobial Zhu et al. (2011)
8	19.96	Anthranilic acid	137	C_7_H_7_NO_2_	3.16	Antiendotoxic Fang et al. (2005)
9	20.37	*trans*-2-Decenoic acid	170	C_10_H_18_O_2_	9.44	–
10	26.16	Tributyl phosphate	266	C_12_H_27_O_4_P	7.69	–
11	31.70	Hexahydro-3-(1-methylethyl)pyrrolo[1,2-a]pyrazine-1,4-dione	210	C_11_H_18_N_2_O_2_	4.9	Antimicrobial, antifungal Yan et al. (2004) Rhee (2004)
12	32.06	Hexahydro-3-(1-methylethyl)pyrrolo[1,2-a]pyrazine-1,4-dione	210	C_11_H_18_N_2_O2	6.84	Antimicrobial, antifungal Borthwick (2012), Campbell et al. (2009) Yan et al. (2004) Rhee (2004)
13	40.12	Hexahydro-3-(phenylmethyl)pyrrolo[1,2-a]pyrazine-1,4-dione	244	C_14_H_16_N_2_O_2_	1.81	Antimicrobial, antifungal Kumar et al. (2013)
					87.52 (Total)	

^a^ RT for retention time in minute

^b^%Pk for percent of peak area

^c^Comment includes bioactivity and references

### Purification of bioactive compounds

The oily yellow residue (2.7 g) was subjected to column chromatography on a silica gel column (Qingdao Haiyang Chemical Co., Ltd., Qingdao, China) pre-equilibrated with dichloromethane and eluted with a gradient solvent system dichloromethane-methanol (v/v, 20:1 to 0:100). Seven fractions were collected and tested for antimicrobial potential with plate inhibitory assay. Fraction 4 (1.1 g), showing inhibitory activity against *Pseudomonas* sp. ZS1, was subjected to silica gel column chromatography and eluted with hexane-dichloromethane (3:1 v/v). Repeated chromatography led to pure compound 1 (10 mg).

### Structure elucidation of bioactive compounds

The structure of the compound 1 was determined using NMR spectroscopy (Bruker DRX 500 NMR instrument, Bruker, Rheinstetten, Germany). CDCl_3_ (Deuterated chloroform) was used as solvent in ^1^H and ^13^C NMR experiments. ^1^H NMR spectra were recorded in CDCl_3_ using tetramethylsilane (TMS) as internal standard at 500 and 400 MHz, ^13^C NMR spectra were recorded at 125 and 100 MHz, chemical shifts are given in parts per million and coupling constants in Hz.

### Determination of minimum inhibitory concentrations (MICs)

To investigate the minimum inhibitory concentration of compound 1 or cyclodipeptide c-(L-Pro-L-Phe), we followed the protocol described Singh-Babak et al. (Singh-Babak et al. 2012). In brief, the compound and ciprofloxacin (Aladdin Industrial Co. Shanghai, China) was subjected to twofold serial dilution from 1025 to 1 µg mL^−1^ in 100 µL of Luria–Bertani (LB) broth (Bertani 1951) using multiwell plate in duplicate. Fresh overnight culture of ZS1 in LB was diluted to a final concentration of 5E−04 OD_600_. The resulting culture of 100 µL was transferred and mixed with twofold serial dilutions of compound or ciprofloxacin. The plate was incubated at 30 °C for 24 h prior to OD measurement. The minimum concentration of the well without bacterial growth was defined as minimum inhibitory concentration (MIC). The MIC of compound 1 and Ciprofloxacin was 32 and 2 µg mL^−1^, respectively.

### Gravimetric analysis of crude oil consumptions

To estimate the consumption of crude oil by *Pseudomonas* sp. ZS1 in presence and absence of *Alcaligenes* sp. CT10, cell mass and crude oil quantity (maximum level was set to 100%) were determined in microbial cultures (i.e., ZS1, CT10, and mixture of ZS1 and CT10) in 180 rpm shake flask at 30 °C containing MS medium supplemented with 1% crude oil. Both cell mass and crude oil mass were determined gravimetrically. In brief, cells were pelleted from 50 mL culture by centrifugation, resuspended in 0.5 mL MS medium, and transferred to filter paper for drying in an oven. Dried filter paper was weighted prior to and after addition of cells. Crude oil mass was determined after removal of cell mass by centrifugation. Oil in supernatant was extracted using hexane that was evaporated prior to weighting. Samples at 36 days were also analyzed using GC–MS and GC analyses.

### Gas chromatography analysis of crude oils in cultures of CT10 and ZS1

The composition of crude oils was analyzed using the GC–MS methodology similar to the analysis of compounds in supernatant of CT10 cultures (see above). To analyze level-changes of individual molecules in crude oils extracted from supernatant of cultures, 1 µL of sample was directly injected into the injection port of gas chromatograph (Shimadzu Co.) equipped with flame ionization detector (FID) and Rtx-5 column (30 m × 0.32 mm, id. with 0.25 µm film thickness) (Restek Co., Bellefonte, PA, USA). The sample injection was made in split mode and the split ratio was 20:1. The temperature of the injection port and detector temperature were set to 280 and 305 °C, respectively. The temperature program was started with an initial temperature at 70 °C and held for 2 min at this temperature, then 25 °C min^−1^ to 140 °C, followed by an additional increase of 3 °C min^−1^ to 240 °C, then 10 °C min^−1^ up to 300 °C, held for 15 min. The total duration of the temperature program was 59.13 min. Nitrogen was used as carrier gas, and its flow rate was 30 mL min^−1^. Hydrogen gas flow rate and air flow rate were 40 and 400 mL min^−1^, respectively. Level of individual compositions was estimated based on the peak area and degradation rate was based on the formula below:

$${\text{DEG}}\% = \left( {{\text{LEVEL}}_{\text{ctl}} - {\text{LEVEL}}_{\text{smp}} } \right)/{\text{LEVEL}}_{\text{ctl}}$$

where DEG% is the rate of degradation, LEVEL_ctl_ and LEVEL_smp_ are compound level in control and in sample, respectively.

## Results

### Analysis of population dynamics in mixed culture derived from oil sludge

**Fig. 1:**
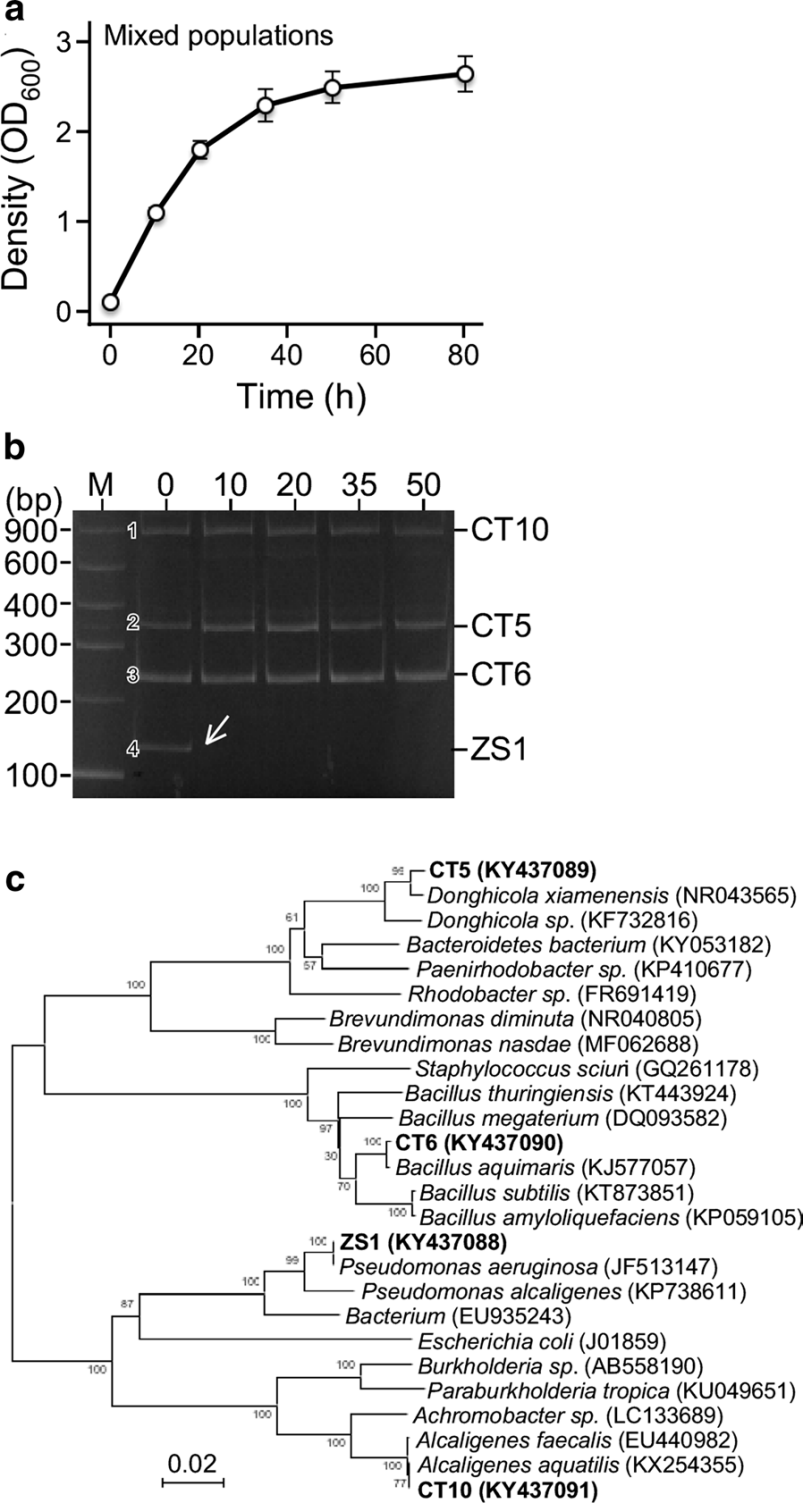
Growth of ZS1 strain is inhibited by other sludge-derived microbes in MS medium supplemented with 2% YE. **a** Growth curve of the sludge-derived mixed culture in MS medium with 2% YE. **b** Dynamic change of microbial populations in mixed culture. Image of mT-RFLP analysis. Four major microbial populations in the initial culture (at 0 h) are numbered. **c** Phylogenetic tree analysis based on 16S rDNA sequences. The tree is built using CLUSTALW and NJPLOT. Sequence accession number of all strains is shown in parentheses

The oil sludge-derived mixed microorganisms were suspended in MS medium and subsequently inoculated into the fresh MS medium supplemented with 2% yeast extract (YE) (see “Materials and methods”). The growth of the mixed culture was monitored by colorimetric methodology (OD_600_) (Fig. 1a). To investigate the microbial population dynamics, total DNA was extracted from the culture at various time points during growth and then subjected to the modified T-RFLP (mT-RFLP) analysis (see “Materials and methods”).

In this analysis, each RFLP fragment would represent a unique microbial population. We found that four populations, namely CT5, CT6, CT10, and ZS1, were present in the initial culture (at 0 h time point). However, 50 h after growth, only three populations CT5, CT6, and CT10 remained (Fig. 1b). Strains from the four major populations were isolated from the initial culture (0 h) based on the mT-RFLP patterns. Analysis of the 16S rDNA sequences indicated that the four strains were *Donghicola* sp. CT5, *Bacillus* sp. CT6, *Alcaligenes* sp. CT10, and *Pseudomonas* sp. ZS1 (Fig. 1c). Of these four strains, *Pseudomonas* sp. ZS1 was previously isolated from the oil sludge (Cheng et al. 2017). Given that all four major strains grew well individually in MS medium supplemented with 2% YE (Additional file 1: Figure S1), this result suggested that ZS1 growth was suppressed by one of the CT5, CT6, and CT10 strains.

### Antagonisms found between the four major populations

**Fig. 2:**
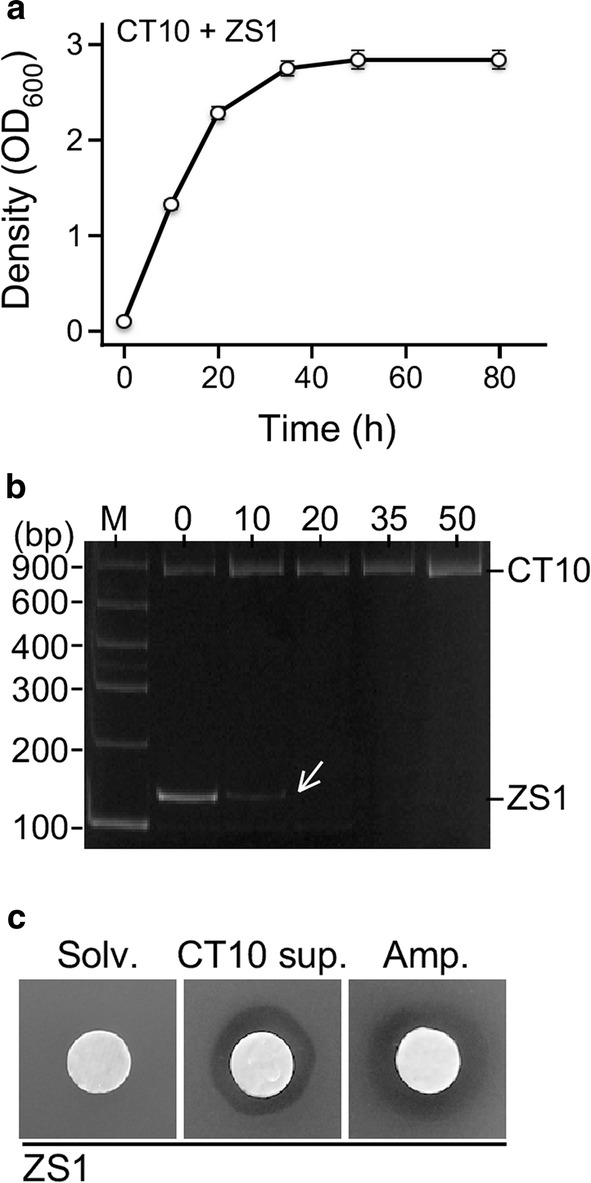
*Alcaligenes* sp. CT10 antagonizes against *Pseudomonas* sp. ZS1. **a** Growth curve of the mixed CT10 and ZS1 cultures. **b** Dynamic change of CT10 and ZS1 populations in mixed culture. Arrow indicates the point that the population diminished. **c** Plate halo assay showing that the growth of ZS1 is inhibited by supernatant extract derived from CT10 cultures

To investigate the potential antagonism between ZS1 and CT5, CT6, or CT10, co-growth analysis was performed (see “Materials and methods”). In the co-growth analysis between ZS1 and CT5 or CT6 using mT-RFLP method to monitor change of populations, we found that ZS1 inhibited the growth of CT5 and CT6, rather than the reverse (Additional file 1: Figure S2). Plate inhibitory assay indicated that this was a result of rhamnolipid (Additional file 1: Figure S2). On the other hand, in the co-growth analysis between ZS1 and CT10, mT-RFLP analysis indicated that ZS1 population failed to growth at 10 h after co-growth (Fig. 2a, b). This was the first time to observe that *Alcaligenes* sp. exhibited antagonistic activity against *Pseudomonas* sp. To investigate whether inhibition factors against ZS1 were secreted into the medium from CT10, supernatant extract of CT10 culture was prepared (see “Materials and methods”). Plate inhibition assay using supernatant extract from CT10 culture on a disc paper indicated that it exhibited apparent inhibitory effect against ZS1 (Fig. 2c). This result indicated that *Alcaligenes* sp. CT10 secreted the unknown factor that antagonized against *Pseudomonas* sp. ZS1.

### Cyclodipeptide c-(L-Pro-L-Phe) from CT10 displays inhibitory activity against ZS1

**Fig. 3:**
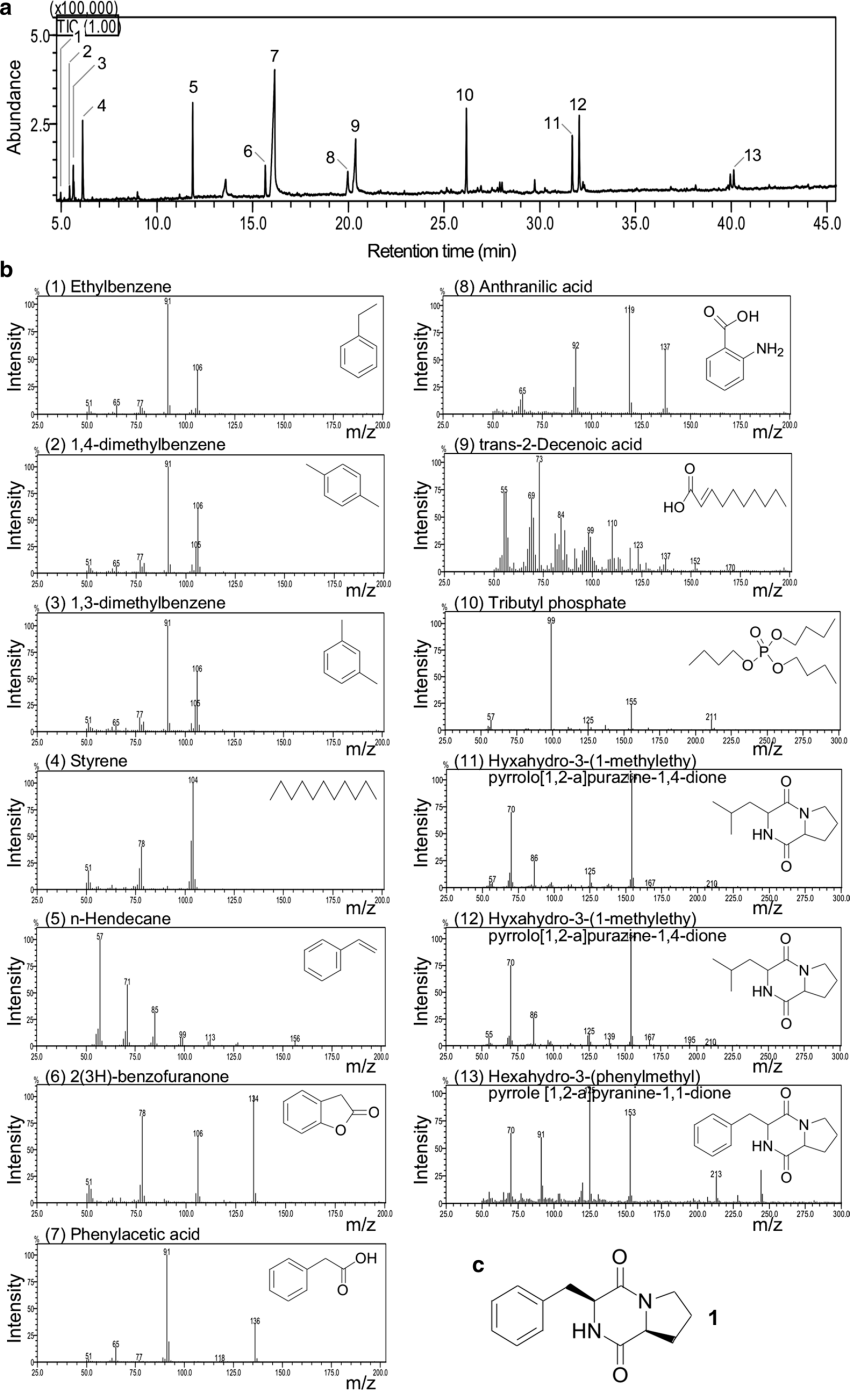
GC-MS analysis of compounds extracted from supernatant of *Alcaligenes* sp. CT10 culture. **a** Total ion chromatograph. Peaks with matched molecules are numbered. **b** MS spectra of individual compounds indicated. **c** An inhibitory compound to ZS1 from CT10. NMR analysis indicates that the compound 1 is the cyclodipeptide c-(L-Pro-L-Phe)

To investigate the potential antagonistic factors against ZS1, supernatant extract derived from CT10 culture was subjected to GC-MS analysis (see “Materials and methods”). The result indicated that 13 major peaks or compounds were detected (Fig. 3a). Of the 13 peaks, peak 11 and 12 represented the same molecule cyclodipeptide c-(Pro-Leu), suggesting that the two stereoisomers c-(D-Pro-L-Leu) and c-(L-Pro-L-Leu) were separated (Fig. 3b). To this end, a number of compounds that were shown to be bioactive such as insecticidal (peak 6, 2(3H)-benzofuranone) (Fan et al. 2008), antiendotoxic (peak 8, anthranilic acid) (Fang et al. 2005), antimicrobial and antifungal (peak 7, phenylacetic acid; peak 11 and 12, hexahydro-3-(1-methylethyl) pyrrolo[1,2-a]pyrazine-1,4-dione; peak 13, hexahydro-3-(phenylmethyl) pyrrolo[1,2-a]pyrazine-1,4-dione) (Fan et al. 2008; Zhu et al. 2011; Kumar et al. 2013; Yan et al. 2004; Rhee 2004) (Table 1). Hexahydro-3-(phenylmethyl) pyrrolo[1,2-a]pyrazine-1,4-dione (peak 13) was cyclodipeptide c-(D-Pro-L-Phe) or c-(L-Pro-L-Phe), which was isolated from *Bacillus* sp. N strain and showed to be inhibitory against *Pseudomonas* sp. at a MIC (minimal inhibitory concentration) of 32–64 µg mL^−1^ (Kumar et al. 2013), suggesting that the antagonistic effect from *Alcaligenes* sp. against *Pseudomonas* sp. was partly attributed to the cyclodipeptides c-(D-Pro-L-Phe) and c-(L-Pro-L-Phe). To test this possibility, we undertook the purification process for the inhibitory activity against *Pseudomonas* sp. ZS1 (see “Materials and methods”). The purified compound was subsequently subjected to ^1^H and ^13^C NMR spectroscopic analysis.

Structure determination of the compound **1** (Fig. 3c): white powder; C_14_H_16_N_2_O_2_; ESI–MS *m/z*: 244 [M + H]^+^; ^1^H NMR (CDCl_3_, 500 MHz) δ_H_: 7.34 (2H, dd, J = 7.5, 9 Hz), 7.26 (1H, t, J = 9 Hz), 7.23 (2H, d, J = 7.5 Hz), 5.88 (1H, S), 4.29 (1H, dd, J = 2.5, 7.5 Hz), 4.05 (1H, t, J = 7.5 Hz), 3.61 (2H, m), 3.58 (1H), 2.80 (1H, dd, J = 10.0, 14.5 Hz), 2.32 (1H, m), 1.99 (1H, m), 1.89 (2H, m); ^13^C NMR (CDCl_3_,125 MHz): 169.5, 165.1, 135.9, 129.2, 129.1, 127.5, 59.1, 56.2, 45.4, 36.8, 28.3, 22.5. Based on the NMR spectroscopic analysis of c-(L-Pro-L-Phe) by Kumar et al. (Kumar et al. 2013), the compound **1** from ZS10 was identified as cyclodipeptide c-(L-Pro-L-Phe), which exhibited a potent inhibitory activity against ZS1 at a MIC of 32 µg mL^−1^ against ZS1 (see “Materials and methods”).

### Gravimetric analysis of oil degradation by ZS1 strain is disrupted by the presence of CT10 strain

**Fig. 4:**
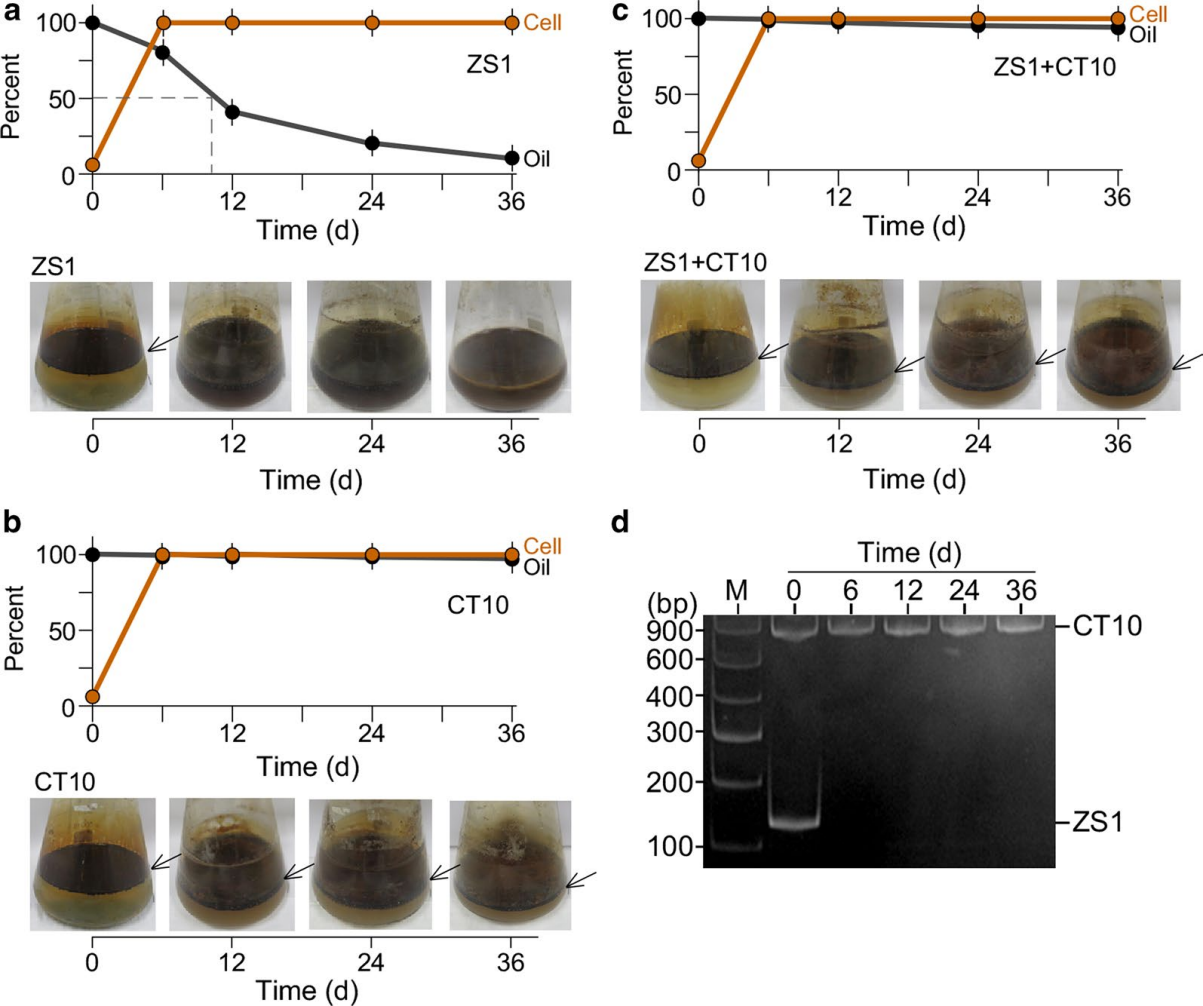
Oil-eating activity of *Pseudomonas* sp. ZS1 is impeded in the presence of *Alcaligenes* sp. CT10. Arrow indicates the presence of floating oil on the surface of cultures. **a** Change of cell mass and crude oil quantity in ZS1 culture. Upper panel shows the percentage of cell mass (Cell) and crude oil mass (Oil) detected in cultures at various time points indicated. Bottom panel shows the presence (with arrow) or absence (without arrow) of floating oil in culture flask. The 50% reduction of crude oil occurs at 10 days after growth. **b** Change of cell mass and crude oil quantity in CT10 culture. The display is identical to **a**. **c** Change of cell mass and crude oil quantity in ZS1 and CT10 mixed culture. The display is identical to **a**. **d** Dynamic change of ZS1 and CT10 populations in mixed culture indicated in (**c**)

To investigate if efficiency of oil degradation by ZS1 strain would be affected in presence of *Alcaligenes* sp. CT10, the oil degradation experiments were performed in MS medium supplemented with 1% crude oil (see “Materials and methods”). Oil residues remained in the medium at various time points during growth was determined gravimetrically by using hexane extraction and weighted after evaporation (see “Materials and methods”). We found that 50% of oils was degraded in ZS1 culture 10 days after growth (Fig. 4a). On the other hand, there was hardly any oil degradation activity detected in culture of *Alcaligenes* sp. CT10 (Fig. 4b). However, oil degradation ability of ZS1 strains was nearly abolished when CT10 strain was present in the culture (Fig. 4c). mT-RFLP analysis confirmed that ZS1 failed to grow in presence of CT10 (Fig. 4d). These results indicated that antagonisms against oil-eating microbes could abolish its oil degradation activity.

### GC–MS analysis of oil degradation by ZS1 strain is impeded by the presence of CT10 strain

**Fig. 5:**
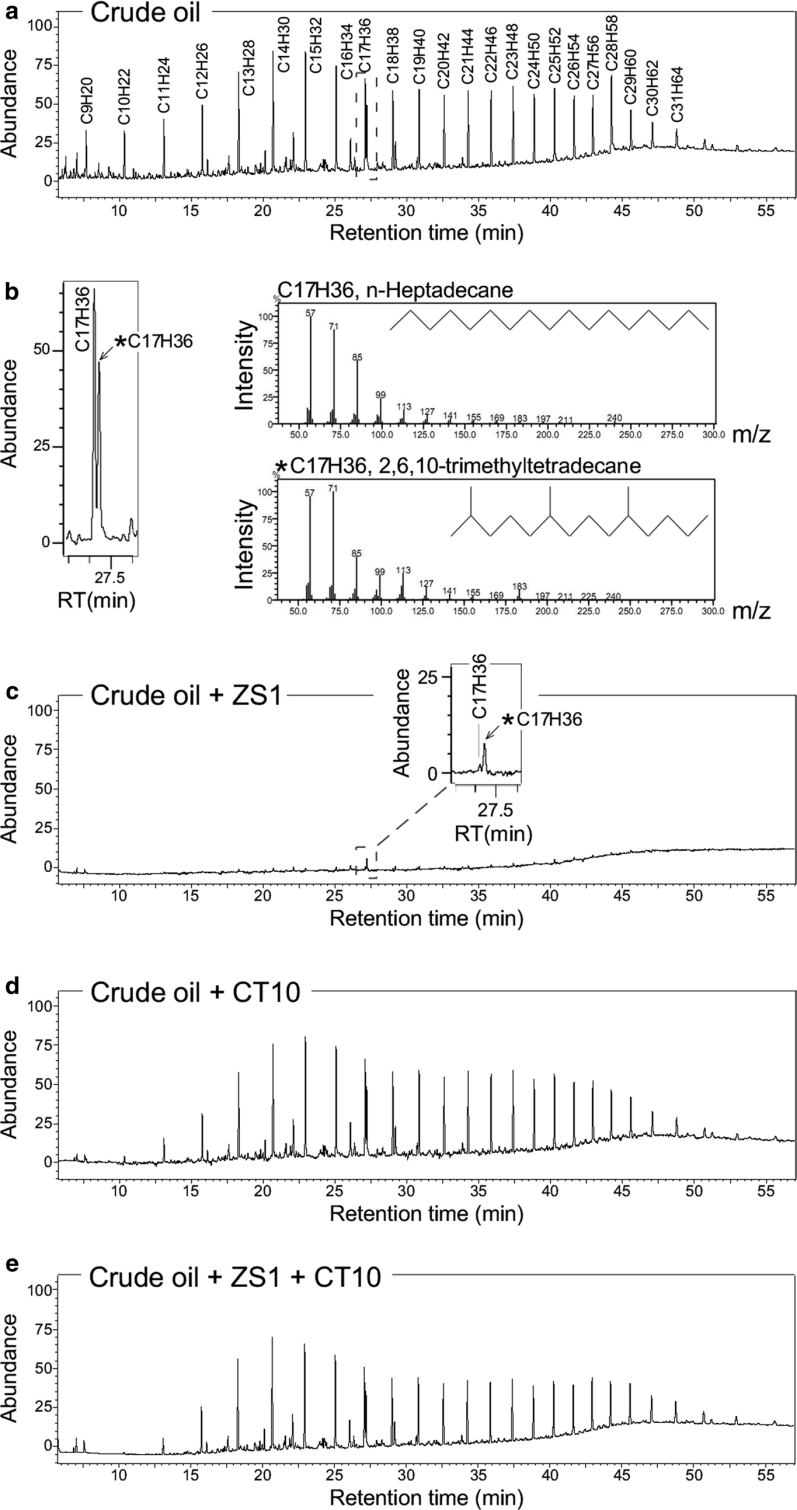
GC analysis of crude oil degradation in culture of *Pseudomonas* sp. ZS1 in presence or absence of *Alcaligenes* sp. CT10. **a** GC analysis of hexane extract derived from medium 36 days after shaking without bacteria. A GC spectrum of crude oil in medium is shown. **b** Linear and branched aliphatic hydrocarbons C_17_H_36_ detected. Left panel shows an enlarged image of the Fig. 5a. MS spectra of the linear (upper right penal) and branched (bottom right panel) C_17_H_36_ are shown. **c** A GC spectrum of oils in ZS1 culture at 36 d after growth. An inset shows the branched C_17_H_36_ but not linear C_17_H_36_ remained noticeable. **d** A GC spectrum of oils in CT10 culture at 36 days after growth. **e** A GC spectrum of oils in mixed CT10 and ZS1 culture at 36 days after growth

Based on the GC–MS analysis, the crude oils used in this study were found to contain 23 linear aliphatic hydrocarbons ranged from C9 to C31 (Additional file 1: Figure S3). Crude oils in supernatant of various cultures 36 days after growth were hexane extracted for GC analysis (see “Materials and methods”). Oils recovered 36 days after incubation in medium without bacteria was used as control for initial levels of various hydrocarbon molecules (Fig. 5a). We noted that a residue of branched aliphatic hydrocarbon *n*-heptadecane present in the crude oil (Fig. 5b). Based on GC analysis, we found that 97.4% of crude oils were degraded in culture of *Pseudomonas* sp. ZS1 36d after growth, though a trace amount of branched hydrocarbon *n*-heptadecane remained to be detected (Fig. 5c, Table 2).

**Table 2:** Degradation of crude oil in cultures of one or both of *Alcaligenes* sp. CT10 and *Pseudomonas* sp. ZS1

LAH (C#)^a^	RT (min)^b^	Ctl (level)^c^	CT10 (level)^d^	CT10/ZS1 (level)^e^	ZS1 (level)^f^	CT10 (%deg)^g^	CT10/ZS1 (%deg)^h^	ZS1 (%deg)^i^
9	7.68	8570	0	0	0	100.00	100.00	100.00
10	10.34	9518	1810	393	0	80.98	95.87	100.00
11	13.09	11,662	4742	3503	0	59.34	69.96	100.00
12	15.76	14,531	9192	9351	0	36.74	35.65	100.00
13	18.29	20,599	17,001	18,330	453	17.47	11.02	97.80
14	20.68	23,975	22,193	22,858	598	7.43	4.66	97.51
15	22.94	26,059	25,468	22,826	664	2.27	12.41	97.45
16	25.07	21,935	21,923	19,366	777	0.05	11.71	96.46
17	27.09	21,009	20,503	17,364	309	2.41	17.35	98.53
18	29.02	16,658	16,654	14,154	0	0.02	15.03	100.00
19	30.85	16,659	16,722	15,411	300	− 0.38	7.49	98.20
20	32.59	16,032	15,933	13,415	592	0.62	16.32	96.31
21	34.26	16,938	17,050	13,738	547	− 0.66	18.89	96.77
22	35.85	16,626	16,535	13,159	378	0.55	20.85	97.73
23	37.38	17,060	17,005	13,042	624	0.32	23.55	96.34
24	38.85	15,283	15,178	11,839	428	0.69	22.53	97.20
25	40.27	15,747	15,757	12,688	658	− 0.06	19.43	95.82
26	41.63	14,552	14,426	11,597	447	0.87	20.31	96.93
27	42.94	13,404	13,273	12,236	532	0.98	8.71	96.03
28	44.23	20,840	11,537	10,560	694	44.64	49.33	96.67
29	45.58	10,534	10,344	10,563	723	1.80	− 0.28	93.14
30	47.08	7432	7411	7377	0	0.28	0.74	100.00
31	48.77	6251	6117	6643	0	2.14	− 6.27	100.00
Total LAH.		361,874	316,774	280,413	8724	12.5	22.5	97.6
Total oil		481,954	428,608	374,615	13,911	11.1	22.3	97.1

^a^LAH (C#) for linear aliphatic hydrocarbons with carbon numbers

^b^RT for retention time in minute

^c^ctl (level) for levels in control

^d^CT10 (level) for levels in *Alcaligenes* sp. culture

^e^CT10/ZS1 (level) for levels in mixed *Alcaligenes* sp. and *Pseudomonas* sp. cultures, respectively

^f^ZS1 (level) for levels in *Pseudomonas* sp. culture

^g^CT10 (%deg) for oil degradation rate in *Alcaligenes* sp. culture

^h^CT10/ZS1 (%deg) for oil degradation rate in mixed *Alcaligenes* sp. and *Pseudomonas* sp. culture

^i^ZS1 (%deg) for oil degradation rate in *Pseudomonas* sp. culture

On the other hand, oils were reduced by 12.5% compared to the control levels in culture of *Alcaligenes* sp. CT10 36d after growth (Fig. 5d, see Table 2). However, degradation rate of some hydrocarbons such as C9, C10, C11, and C28 was high (degradation rate > 35%). In a mixed culture of ZS1 and CT10, we found that 22.5% of total oils were degraded 36d after growth (Fig. 5e, see Table 2), much lower than that of 97.6% degradation in ZS1 culture, though a bit higher than that of 12.5% in CT10 culture. These results were in agreement with the gravimetric analysis that crude-oil degradation ability of *Pseudomonas* sp. ZS1 strain could be inhibited in presence of *Alcaligenes* sp. CT10.

## Discussion

Enhanced bioremediation is believed to be a useful method for oil pollutant cleanup (Patowary et al. 2016; El-Bestawy et al. 2014). However, there are limitations (Díaz-Ramírez et al. 2003; Venosa and Zhu 2003). Physical and chemical conditions are known to affect the growth of the oil-eating microorganisms at pollutant sites. In this study, we show that biotic factors such as antagonistic species can also influence the growth of the oil-eating microorganisms (see Figs. 4, 5). Hence, oil degradation during enhanced bioremediation can be complicated by not only physical and chemical factors, but also biological factors.

We have previously screened for biosurfactant-producing microorganisms using the mT-RFLP methodology to monitor the enrichment under selective growth conditions. In this study, we show that by using this method, antagonism between microbes is readily detected (see Figs. 1, 2). All major populations observed in mixed culture of the oil sludge-originating microorganisms are found to be involved in one of the antagonistic interactions, implying that antagonism between microbes is not ignorable in environmental niches.

Biosurfactant rhamnolipid is known to inhibit bacteria such as *Serratia marcescens*, *Enterobacter aerogenes*, and *Klebsiella pneumoniae* (Haba et al. 2003). In this study, we show that rhamnolipid produced by *Pseudomonas* sp. ZS1 inhibits the growth of *Donghicola* sp. CT5 and *Bacillus* sp. CT6 (see Additional file 1: Figure S2). It is possible that in a bacterial consortium for bioremediation (Patowary et al. 2016; El-Bestawy et al. 2014), the growth of oil-eating microorganisms could be inhibited by other biosurfactant-producing microbes. In fact, it has been observed that microbial populations change during bioremediation (MacNaughton et al. 1999). Hence, real-time monitoring the change of microbial populations during bioremediation would permits rapid intervention for improving oil-eating bacterial growth and thus increasing the efficiency of oil pollutant cleanup.

In this study, we show that *Alcaligenes* sp. exhibits antagonistic activity against *Pseudomonas* sp. (see Fig. 2). Based on the GC–MS analysis, a number of bioactive compounds are found to be produced by CT10 (see Table 1). In particular, we have purified the compound 1, known as cyclodipeptide c-(L-Pro-L-Phe) that shows a potent inhibitory activity against ZS1 at a MIC of 32 µg mL^−1^.

Cyclodipeptides (CDPs) or 2,5-diketopiperazines (DKPs) are the smallest cyclic peptides that widely spread in nature as secondary functional metabolites or side products of protein metabolism in microorganisms, plants, and animals (Borthwick 2012; Prasad 1995). CDPs are primarily synthesized by the non-ribosomal peptide synthetases (Schwarzer et al. 2003) and cyclodipeptide synthases (Gondry et al. 2009) in microorganisms. They often serve as precursors for modification with various tailoring enzymes that result diverse compounds with numerous bioactivities such as thaxtomin A and gliotoxin. Thaxtomin A is derived from hydroxylation of precursor c-(L-Trp-L-Phe) (Healy et al. 2002), whereas gliotoxin is generated through oxidation, sulfurization, and methylation of precursor c-(L-Phe-L-Ser) (Gardiner and Howlett 2005). Thus, CDPs have shown great potential for new drug development (Borthwick 2012).

Holden et al. (1999) have proposed that CDPs interfere quorum sensing signals in bacteria and hence affect bacterial growth. However, this ideal is challenged by Campbell et al. (2009) whom have shown that none of the CDPs tested exhibit activation or inhibition of quorum sensing signals. Hence, the mechanisms for CDPs to inhibit bacterial growth remain elucidation.

Based on GC analysis, we find that degradation rate of crude oil by *Pseudomonas* sp. ZS1 reaches as high as 97.6% (see Fig. 5, Table 2) in 36d. However, when *Alcaligenes* sp. CT10 is present, the degradation rate reduces by 4.3-fold (degradation rate of 22.5% vs. 97.6%). Degradation of selected hydrocarbons such as C9, C10, C11, and C28 by CT10 is observed, suggesting a complex of hydrocarbon degradation by various environmental microorganisms.

## Additional file

**Additional file 1: Figure S1.** Growth curve analysis of four strains isolated from oil-sludge. **Figure S2.** *Pseudomonas* sp. ZS1 antagonizes against *Donghicola* sp. CT5 and *Bacillus* sp. CT6. **Figure S3.** GC–MS analysis of crude oil compositions used in this study.

## Abbreviations

GC–MS: gas chromatography coupled with mass spectrometry
mT-RFLP: modified terminal-labeled restriction fragment length polymorphism
NMR: nuclear magnetic resonance
