# Supplementary material for: Petroleum degradation by Pseudomonas sp. ZS1 is impeded in the presence of antagonist Alcaligenes sp. CT10
Source: AMB Express. 2018 May 28;8:88. doi: 10.1186/s13568-018-0620-5 (PMC5972140; doi:10.1186/s13568-018-0620-5)

Additional File 1: Figure S1. Growth curve analysis of four strains isolated from oil-sludge. Growth curves of (A) *Alcaligenes* sp. CT10, (B) *Donghicola* sp. CT5, (C) *Bacillus* sp. CT6, and (D) *Pseudomonas* sp. ZS1 in MS medium supplemented with 2% YE.

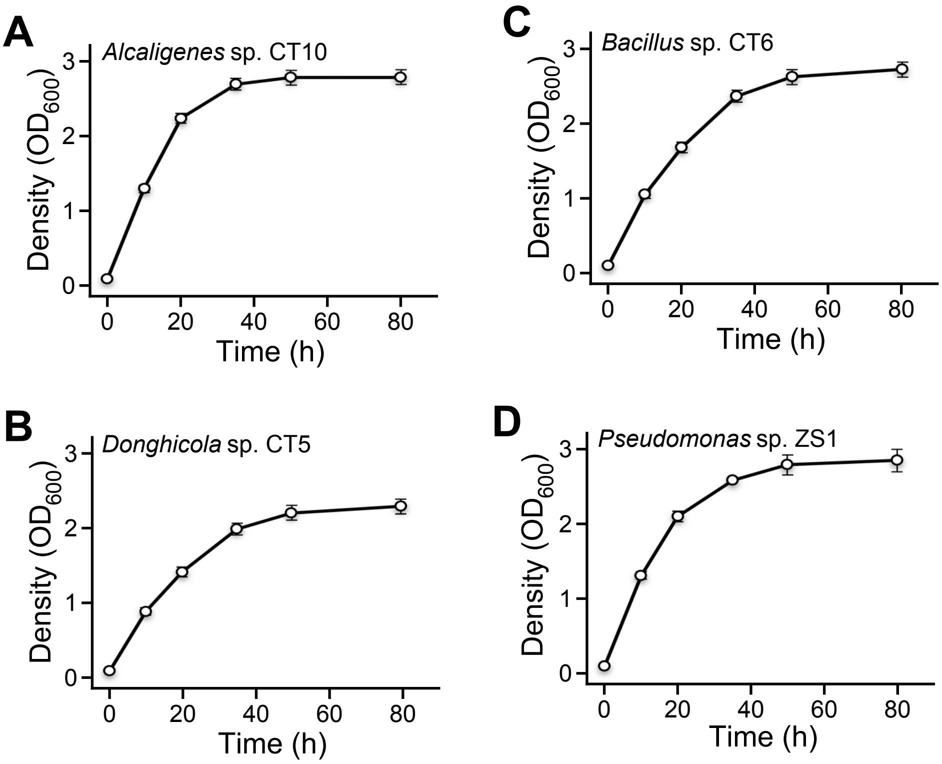

Additional File 1: Figure S2. *Pseudomonas* sp. ZS1 antagonizes against *Donghicola* sp. CT5 and *Bacillus* sp. CT6. (A) Growth curve of the mixed ZS1 and CT5 cultures. (B) Dynamic change of ZS1 and CT5 populations in mixed culture. Arrow indicates the point that the population diminished. (C) Plate halo assay showing that the growth of CT5 is inhibited by rhamnolipid produced by ZS1. (D) Growth curve of the mixed ZS1 and CT6 cultures. (E) Dynamic change of ZS1 and CT6 populations in mixed culture. (F) Plate halo assay showing that the growth of CT6 is inhibited by rhamnolipid produced by ZS1.

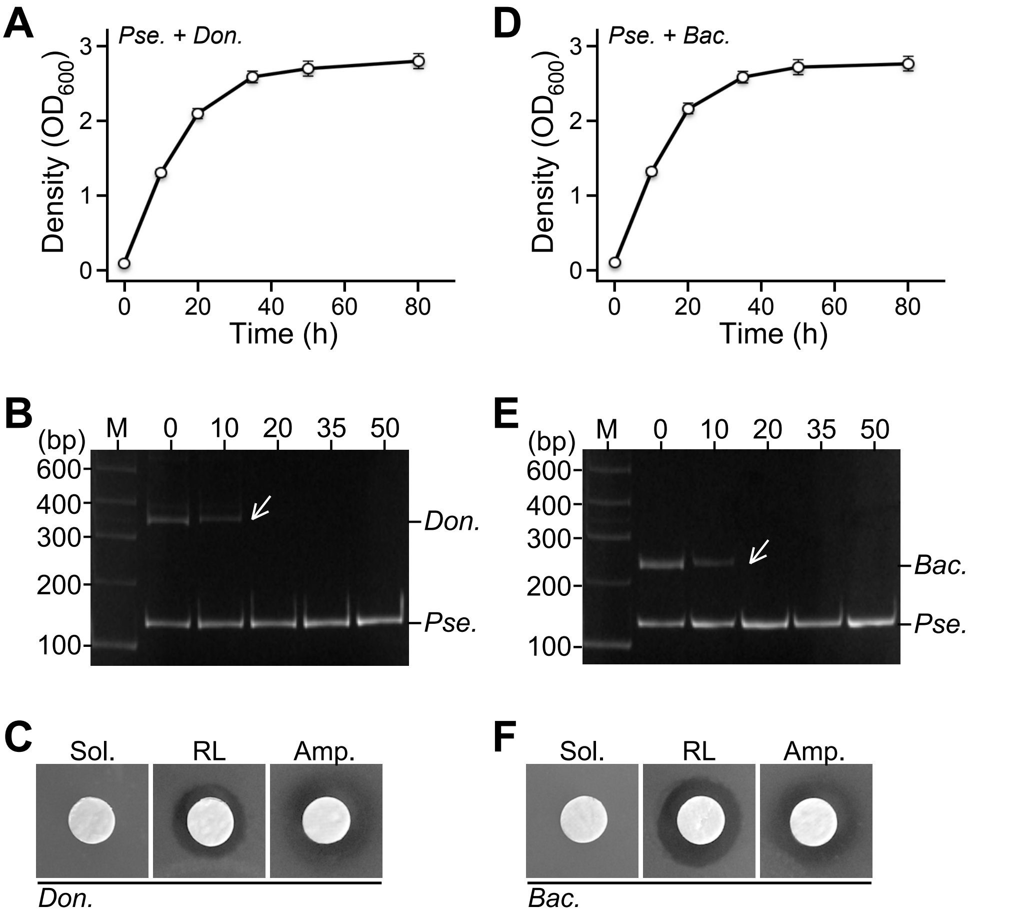

Additional File 1: Figure S3. GC-MS analysis of crude oil compositions used in this study. (A) Total ion chromatogram of the crude oil. (B) Major molecules observed in the crude oil.

A

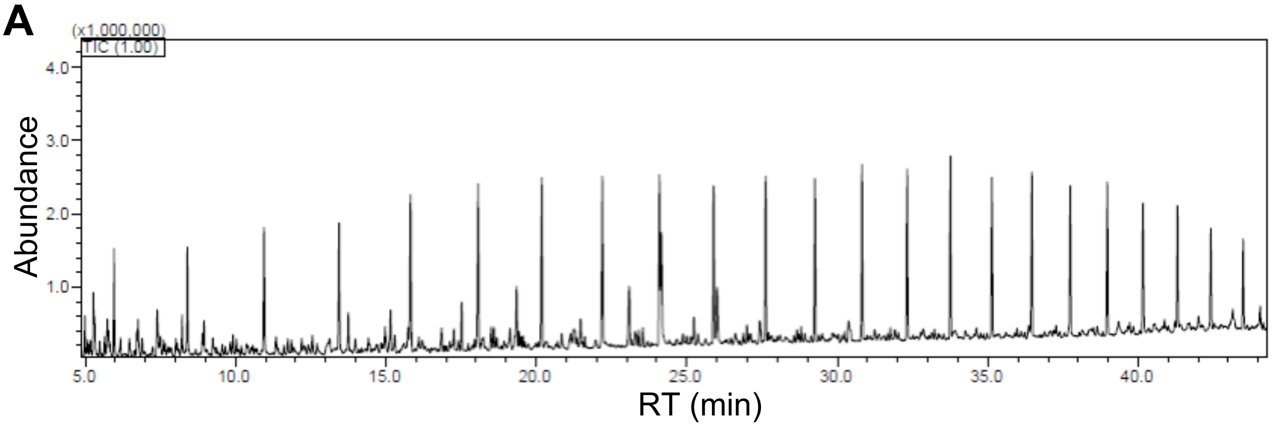

B

Oil composition (carbon no.)	Retention time	Compound Name	Formula	M.W	Molecular structure
9	7.68	n-Nonane	C_9H_{20}	128	
10	10.34	Decane	$C_{10}H_{22}$	142	
11	13.09	n-Hendecane	$C_{11}H_{24}$	156	
12	15.76	Dodecane	$C_{12}H_{26}$	170	
13	18.29	n-Tridecane	$C_{13}H_{28}$	184	
14	20.68	Tetradecane	$C_{14}H_{30}$	198	
15	22.94	n-Pentadecane	$C_{15}H_{32}$	212	
16	25.07	n-Hexadecane	$C_{16}H_{34}$	226	
17	27.09	n-Heptadecane	$C_{17}H_{36}$	240	
18	29.02	Octadecane	$C_{18}H_{38}$	254	
19	30.85	n-Nonadecane	$C_{19}H_{40}$	268	
20	32.59	n-Eicosane	$C_{20}H_{42}$	282	
21	34.26	n-Heneicosane	$C_{21}H_{44}$	296	
22	35.85	n-Docosane	$C_{22}H_{46}$	310	
23	37.38	n-Tricosane	$C_{23}H_{48}$	324	
24	38.85	n-Tetracosane	$C_{24}H_{50}$	338	
25	40.27	n-Pentacosane	$C_{25}H_{52}$	352	
26	41.63	n-Hexacosane	$C_{26}H_{54}$	366	
27	42.94	n-Heptacosane	$C_{27}H_{56}$	380	
28	44.23	n-Octacosane	$C_{28}H_{58}$	394	
29	45.58	n-Nonacosane	$C_{29}H_{60}$	408	
30	47.08	n-Triacontane	$C_{30}H_{62}$	422	
31	48.77	n-Hentriacontane	$C_{31}H_{64}$	436	

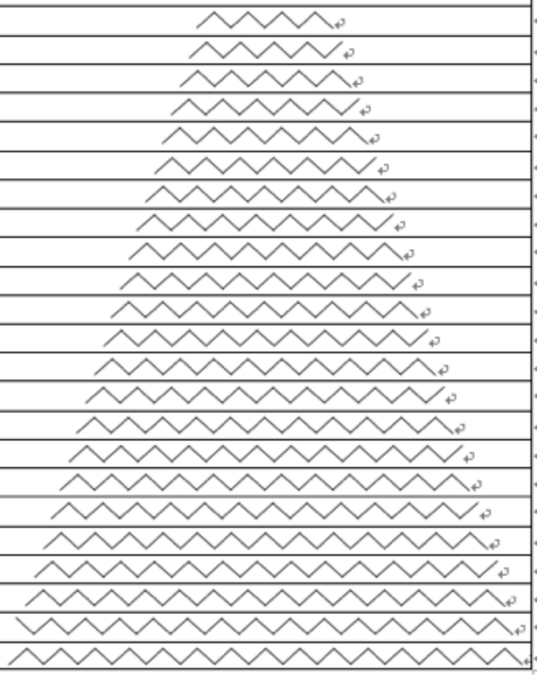

Supplement: Supplementary file 1 — Additional file 1: Figure S1. Growth curve analysis of four strains isolated from oil-sludge. Figure S2. Pseudomonas sp. ZS1 antagonizes against Donghicola sp. CT5 and Bacillus sp. CT6. Figure S3. GC–MS analysis of crude oil compositions used in this study. [file 13568_2018_620_MOESM1_ESM.pdf]
